# Dysphagia Lusoria: An Elusive Case of Dysphagia Caused by a Rare Embryonic Vascular Sling

**DOI:** 10.7759/cureus.89468

**Published:** 2025-08-06

**Authors:** Andrew J Shychuk, Anil Thomas

**Affiliations:** 1 Internal Medicine, University of Florida College of Medicine, Gainesville, USA; 2 Hospital Medicine, Malcom Randall Veterans Affairs Medical Center, Gainesville, USA; 3 Internal Medicine, Malcom Randall Veterans Affairs Medical Center, Gainesville, USA

**Keywords:** dysphagia, dysphagia in elderly, dysphagia surgery, progressive dysphagia, rare cause of dysphagia, sarcopenic dysphagia, vascular dysphagia

## Abstract

Dysphagia lusoria is an uncommon cause of dysphagia with an increasing incidence with age. It is unknown why individuals with dysphagia lusoria typically remain asymptomatic until older adulthood, but some theorize that it could be related to physiologic and anatomical changes that occur with the aging process, such as increased esophageal rigidity and stiffening of vascular walls with atherosclerosis, that make the compression from these congenital aberrations more impactful. While uncommon, it is also likely underrecognized due to its being diagnostically challenging to identify. Moreover, dysphagia may be inappropriately ascribed to being a normal part of aging or ascribed to other coexisting comorbidities when in actuality the cause is multifactorial. Given that different causes of dysphagia warrant distinct interventions, it is important for clinicians to have awareness of unusual or uncommon causes of dysphagia to allow for early detection and intervention to prevent the significant morbidity and mortality that can result. We present a case of dysphagia lusoria in a medically complex older adult male patient admitted for otherwise non-medical care. Due to recognition of the need for multidisciplinary evaluation of complex dysphagia, a timely diagnosis was made, allowing for early intervention with nonsurgical management. The case highlights the need for an integrated approach with close follow-up by the surgical team and speech-language pathology as part of the pathway to ensure best management practice prior to the development of complications.

## Introduction

In the adult population, there is an estimated annual incidence of 25 per 100,000 for dysphagia, with a higher incidence among adult males compared to females [1-2]. While the incidence of dysphagia increases with age, it should not be attributed to the normal aging process, and incidence may be underestimated as individuals may be less likely to report symptoms of dysphagia until they are severe in nature [3]. With increasing incidence, especially after the seventh decade of life, onset is generally insidious with a subjective sensation of difficulty or abnormal swallowing. Individuals experiencing mild dysphagia often compensate with longer eating times due to more extensive mastication or consume liquids with food to help it pass [4]. Dysphagia is notably separate from other issues, such as odynophagia, which is pain with swallowing, and globus sensation, which is a nonpainful sensation of a lump or tightness in the pharyngeal location specifically [5]. Dysphagia can result from a plethora of underlying etiologies, broadly resulting from anatomic or functional causes, and can be primary oropharyngeal or esophageal in location. Common coexisting symptoms with dysphagia may include heartburn, weight loss, hematemesis, anemia, regurgitation of food, and respiratory issues. Depending on the etiology of the dysphagia, it may occur with solid, liquid, or both and may be progressive or intermittent. Given the potential morbidity and mortality that results from dysphagia, including but not limited to weight loss and malnutrition [6], aspiration leading to pneumonia or pneumonitis, and food bolus impaction, it is important for early evaluation into the underlying cause of dysphagia in order to intervene timely enough to prevent complications [7]. Often, careful attention to clinical history and evaluation can enlighten the clinician as to the origin of the dysphagia. Rarer causes, such as dysphagia lusoria with a prevalence of 0.5% to 1.8% may be underrecognized due to a lack of awareness as an entity or due to specialized diagnostic testing needed to identify it [6-9]. A congenital anomaly related to the aberrant right subclavian artery overriding the esophagus, leading to dysphagia from extrinsic compression, dysphagia lusoria was first described in the literature in 1794 by David Bayford, with the name derived from the Latin phrase lusus naturae, meaning “freak of nature” [9]. It is unknown why individuals with dysphagia lusoria typically remain asymptomatic until older adulthood; however, some hypothesize that there could be some contribution related to physiologic and anatomical changes that occur with the aging process, such as increased esophageal rigidity and stiffening of vascular walls with atherosclerosis [9], that make the compression from these congenital aberrations more impactful. As patients may commonly present with early signs of dysphagia in the outpatient clinical setting, it is imperative for clinicians to have a broad working knowledge of causes of dysphagia, however rare, along with familiarity with the specific diagnostic workups needed to distinguish them. We present a patient with complex medical comorbidities with undiagnosed dysphagia lusoria with the aim of broadening awareness of the condition and informing on the diagnostic and multidisciplinary approach to identification and management of dysphagia lusoria.

## Case presentation

An older adult man in his 70s, under the care of an inpatient psychiatric service, developed worsening symptoms of dysphagia. Given his complex medical history, the internal medicine team was consulted for evaluation. The patient’s medical history was notable for gastroesophageal reflux disease, Barrett’s esophagus, atrial fibrillation, history of stroke, depression, cervical spondylosis, alcohol use disorder, and tobacco use disorder. He had been experiencing long-standing intermittent symptoms of difficulty with swallowing, but within the last two months, developed food bolus impaction specifically in the right side of his throat. Consequently, it started necessitating that he perform maneuvers to push food down, and he consumed a modified diet of soft and bite-sized solids. The patient reported that as symptoms progressed, he experienced dysphagia, especially with solid food, but did not have odynophagia. As a result of difficulty with oral intake, he had experienced unintentional weight loss over the last couple of years. 

**Figure 1:**
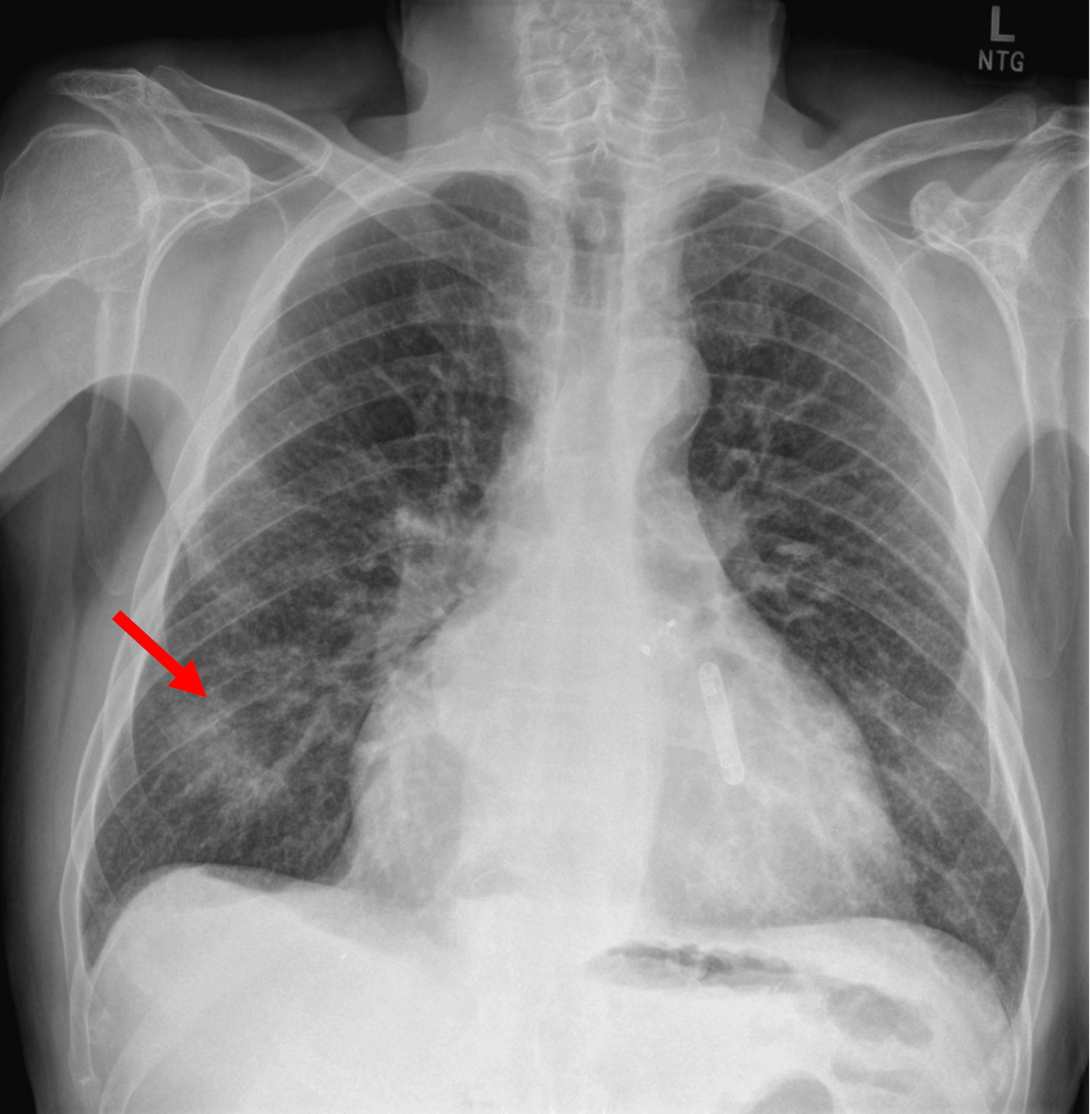
Chest X-ray with the red arrow denoting area of aspiration in the right lower lobe.

**Figure 2:**
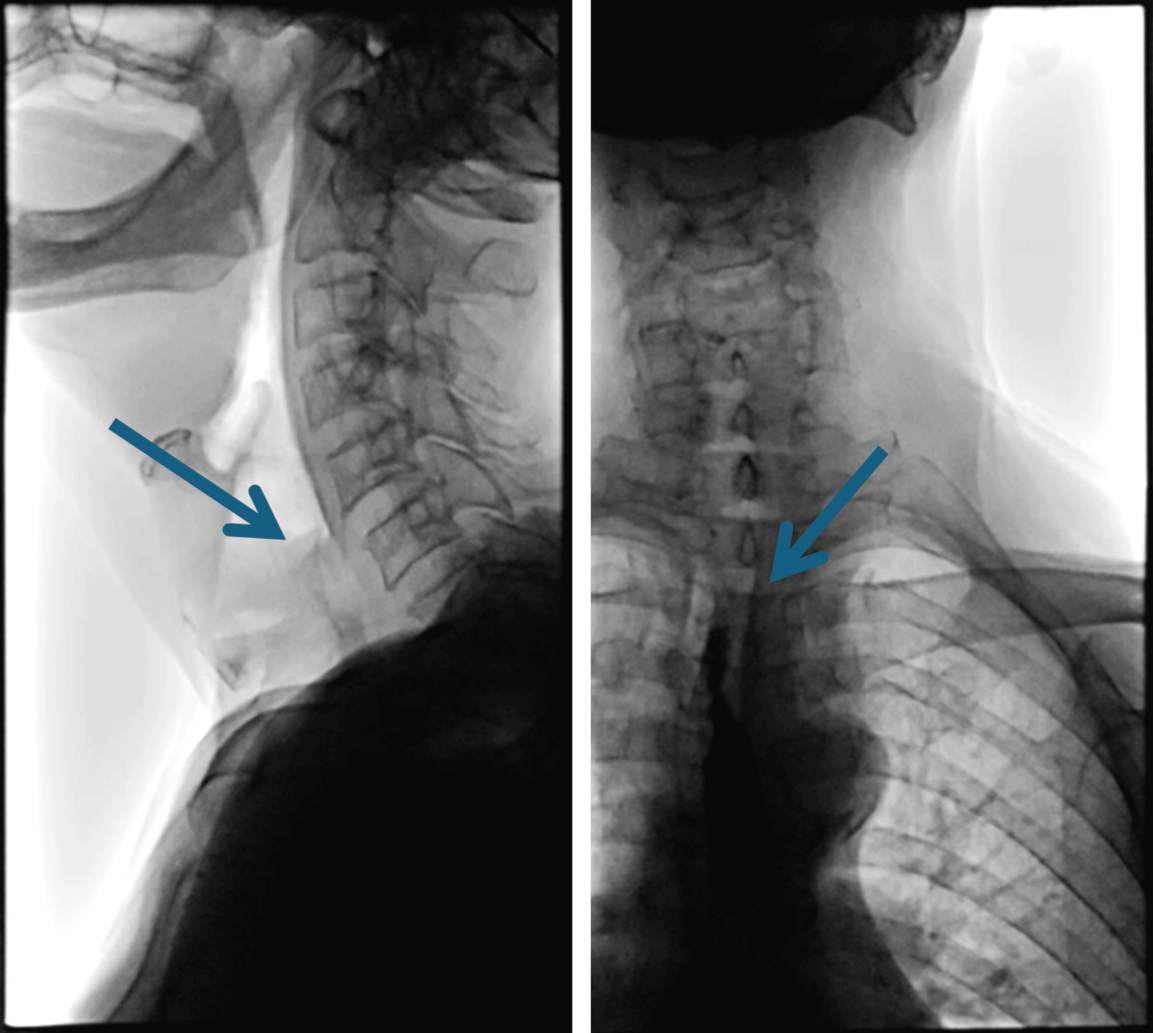
Barium swallow study with video fluoroscopy demonstrating aspiration of thin liquids (blue arrow).

An initial evaluation with a plain film X-ray demonstrated right lower lobe infiltrate (Figure 1). The speech, language, and pathology (SLP) service subsequently evaluated him bedside, and he demonstrated intermittent overt signs and symptoms of aspiration with thin liquids and endorsed globus sensation with solid food intake. As such, they recommended a modified diet with the need for a follow-up barium swallow study with video fluoroscopy, which redemonstrated signs of aspiration of liquids (Figure 2). 

**Figure 3:**
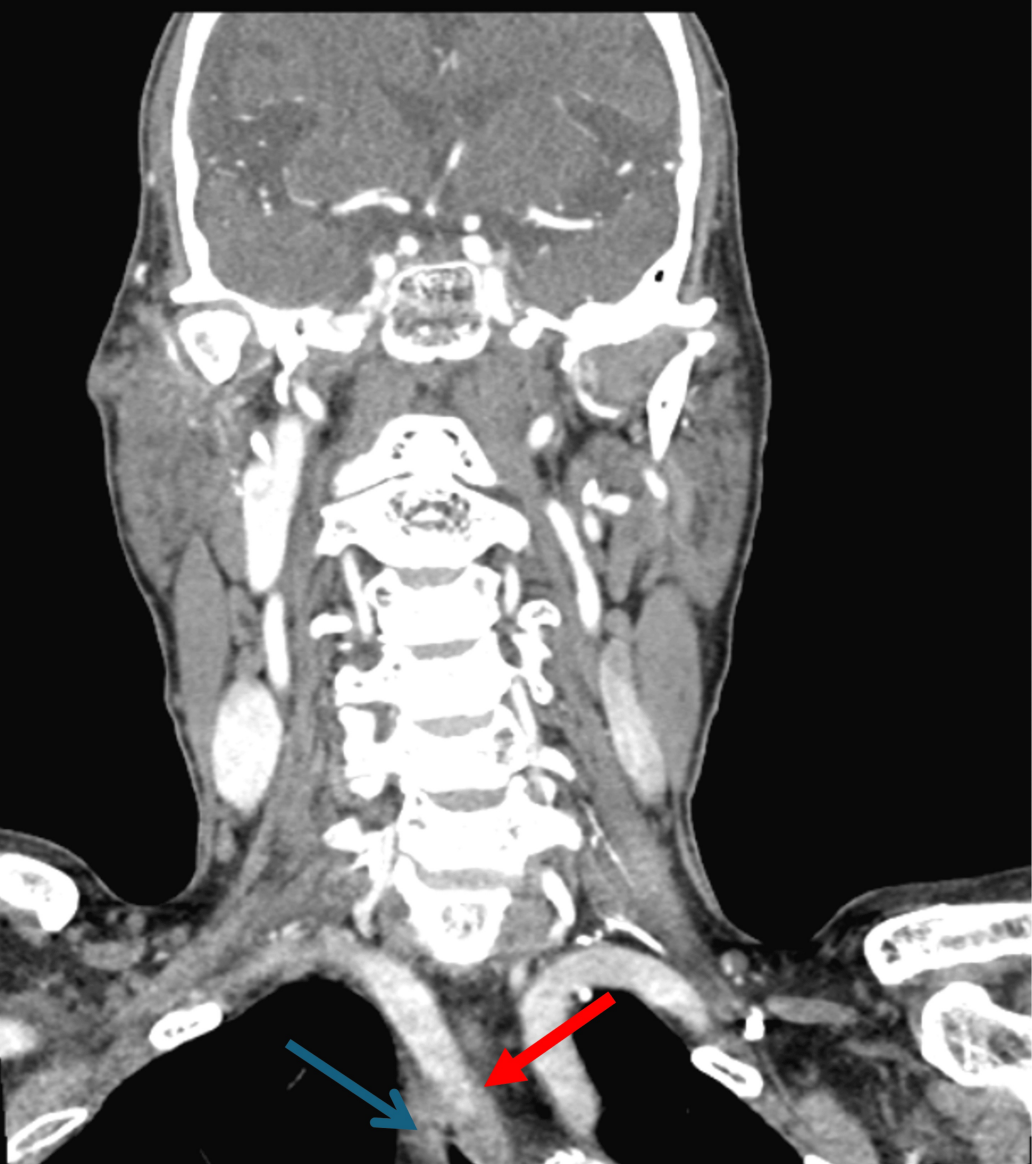
Coronal view of the CT scan of the head and neck with the aberrant right subclavian artery (red arrow) overriding the esophagus (blue arrow).

**Figure 4:**
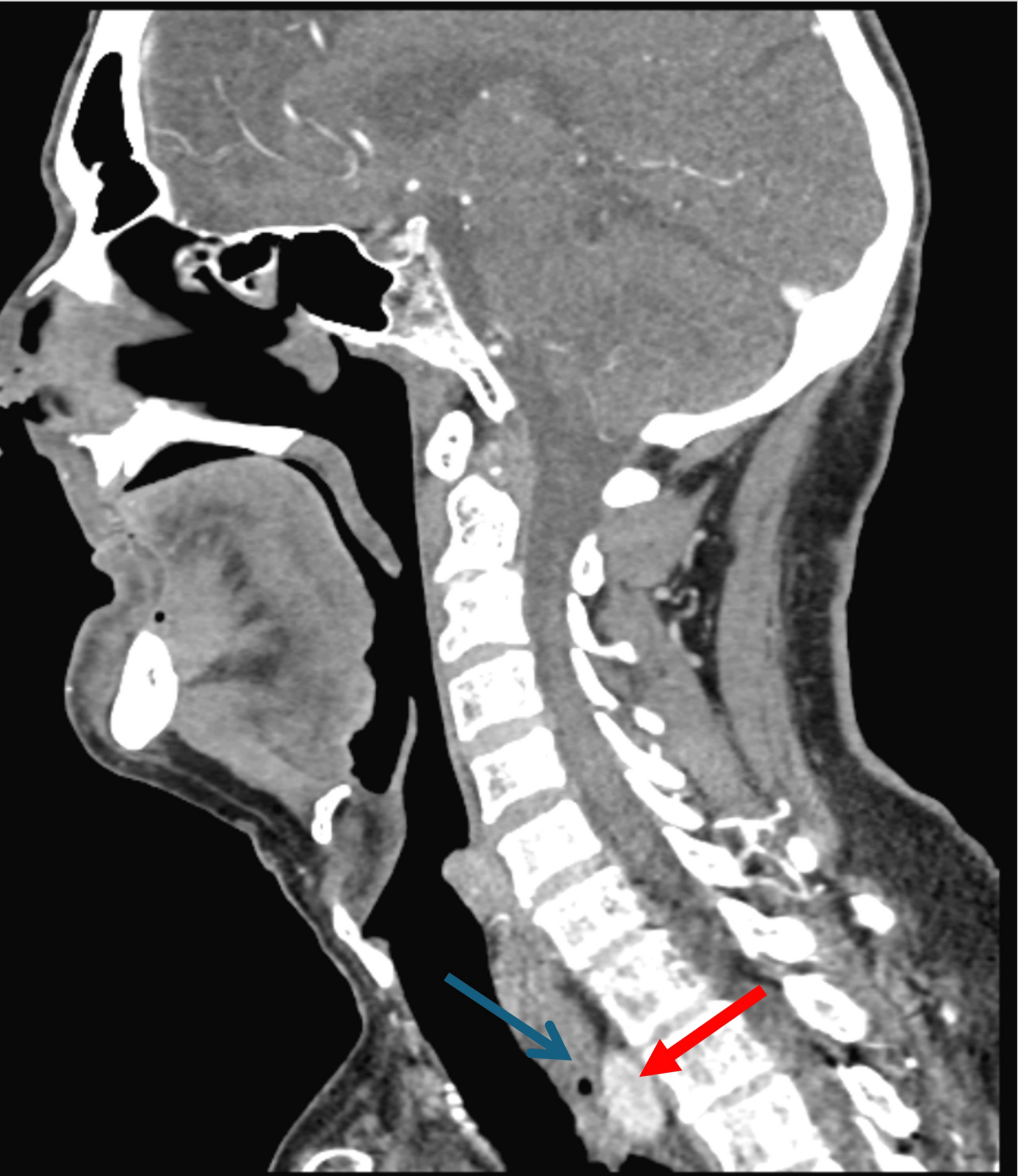
Sagittal view of the CT scan of the head and neck with the aberrant right subclavian artery (red arrow) overriding the esophagus (blue arrow).

**Figure 5:**
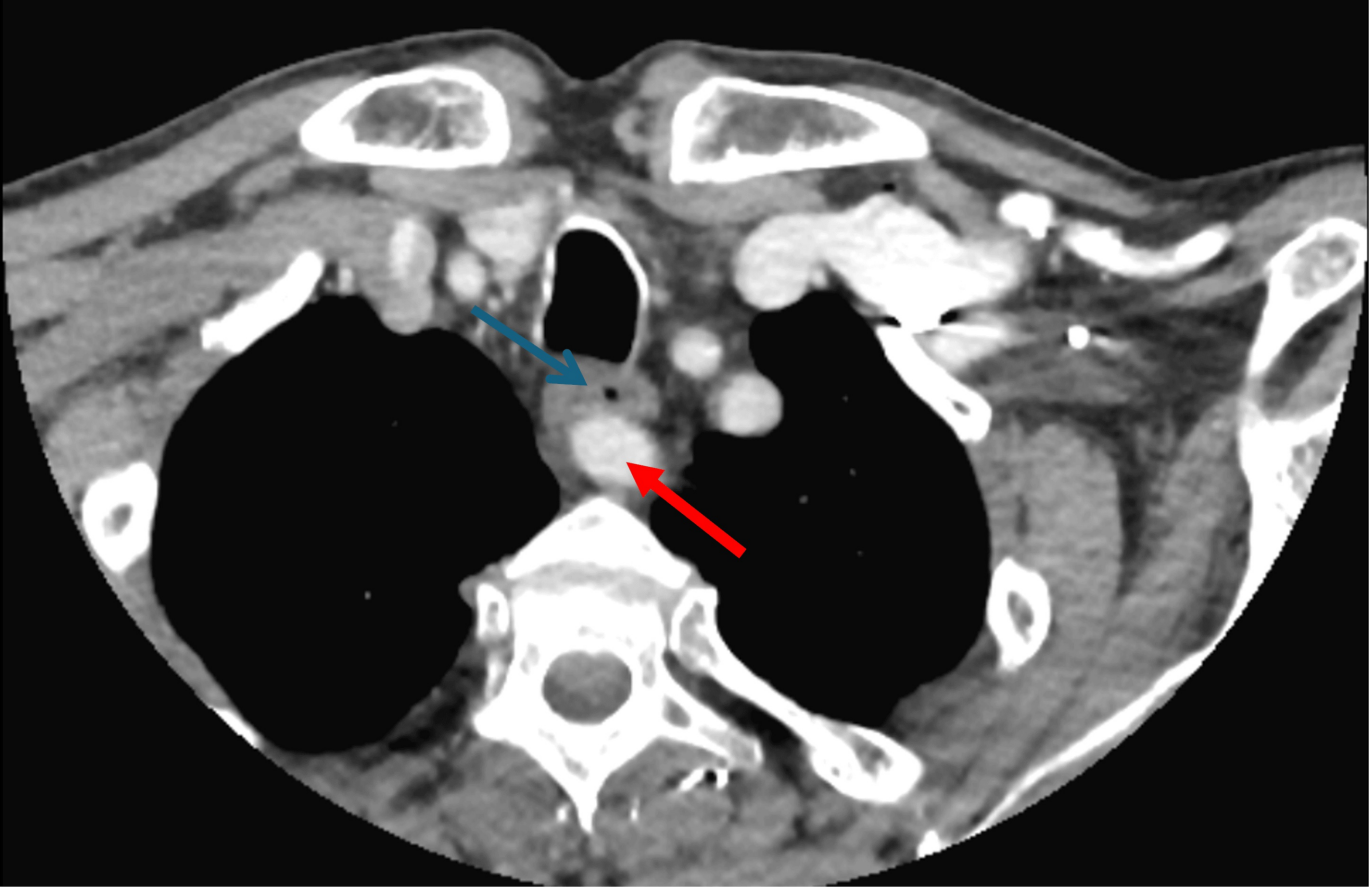
Axial view of the CT head and neck with the aberrant right subclavian artery (red arrow) overriding the esophagus (blue arrow).

Additional imaging with CT with and without contrast was successively performed to further investigate an anatomic cause for dysphagia. While CT did not show any sign of pharyngeal, laryngeal, or esophageal mass, there was demonstration of the right subclavian artery with the upper esophagus centered between the trachea and aberrant vessel, consistent with an embryologic defect of the aortic arch vasculature causing a vascular sling and resulting in a condition known as dysphagia lusoria (Figures 3-5). 

Cardiothoracic surgery was consulted for evaluation of the possible need for surgical intervention. Given the intermittent nature of the symptoms, which were somewhat improved after SLP modifications, advanced age, and high-risk comorbidities, surgery was recommended, along with continued conservative management of dysphagia and outpatient follow-up with both surgery and SLP service to determine the need for intervention at a later date.

The patient remained hospitalized for psychiatric treatment for an additional period of time, during which the medicine consult team continued to follow, and the SLP service continued working on swallowing modification strategies. He exhibited good technique and was not seen to have any significant episodes of food bolus impaction or aspiration with changes. He was referred to follow up with the outpatient cardiothoracic service, but has not yet been seen in follow-up by the time of this manuscript.

## Discussion

Dysphagia is a common condition with increasing incidence in the adult population with each decade of life added [1-8]. As such, it is easy for it to be considered unsubstantiated as a normal process of aging, with full evaluation of the underlying process foregone until symptoms become severe, leading to episodes of choking, aspiration, or pneumonia, and substantial weight loss and malnutrition have already resulted. Given the complex process involved in mastication and swallowing, along with the fact that more patients are living longer with chronic disease, with variability in complications, a thorough workup into the underlying etiology is needed, especially given that common and uncommon causes can present identically but may require substantially different treatment and management.

Dysphagia lusoria results from a rare congenital vascular anomaly with an aberrant right subclavian artery [9-11]. While the average age of most individuals who present with symptomatology is around 50 years of age [12], in severe cases, some may present early in life during the neonatal period or in childhood. Conversely, those with milder symptoms may not be identified until a more advanced age. As dysphagia lusoria is reasonably uncommon, with an estimated prevalence of 0.5% to 1.8% [9-10,13], it may not typically be at the forefront of even the most astute clinician’s or radiologist’s differential, as the likelihood is they have not seen or thought of it since their undergraduate medical training. Apart from dysphagia, esophageal reflux and globus sensation are the other common nonspecific symptoms patients may experience [11]. Furthermore, as with the aforementioned patient, patients may have other underlying conditions that in and of themselves can impair chewing or swallowing [6-7], leading to premature closure of diagnosis prior to considering other things as contributory. Therefore, if the degree of dysfunction is out of proportion to the known condition, one must look further to clarify conditions that are multifactorial in etiology.

While dysphagia lusoria is generally uncommon, it is appropriate to have a lower threshold to rule it out in patients with known cardiovascular or chromosomal abnormalities, which may predispose them to this condition. The initial diagnostic modality of choice is the barium esophagram [9, 11], as this may provide a noninvasive view of esophageal anatomy as well as any functional impact on swallowing. Typical findings of dysphagia lusoria identified on barium esophagram are described as the “bayonet sign” due to characteristic flattening of the esophagus as it is positioned between the trachea anteriorly and the aberrant right subclavian artery posteriorly [11, 14]. As concerns for dysphagia lusoria are identified, it should be investigated further with either a CT angiogram or a magnetic resonance angiogram, which will be particularly useful if additional clarification of the anatomy is needed to plan for surgical intervention [9, 11, 14]. Physical exam is largely unremarkable in identifying the condition, as is the case with evaluation with esophageoduodenoscopy (EGD) [9,15]. Although when EGD is performed with ultrasonography (EUS), it can help in confirming that there is a vascular component locally if compression is visualized endoscopically [9]. Furthermore, some endoscopists will employ EGD with a functional luminal imaging probe (FLIP) that uses a balloon with a probe that allows for measurement of distensibility and motility data. This may allow for differentiation of contributing conditions when dysphagia may be multifactorial in order to tailor treatment [16].

In patients with mild symptoms, treatment with lifestyle modifications such as chewing food fully, eating more slowly, sipping liquids, and taking smaller bites may be sufficient to reduce the likelihood of food bolus impaction or aspiration [9, 11, 14]. However, for those experiencing severe symptoms or individuals who are unable to be managed well enough with conservative measures, surgical intervention may be recommended eventually [9, 11-12]. This can be done by dilation or widening of the esophagus endoscopically, which may be repeated as needed if symptoms return [12-14]. In severe cases, dysphagia lusoria is managed by removal of the aberrant artery with reconstruction and repositioning to the right side of the aortic arch by cardiothoracic surgery [9, 14, 17-20]. Surgery is generally a last resort in severely symptomatic patients, as it can run the risk of disruption of blood flow to extremities and the vertebral artery, leading to serious consequences, which may be a major risk consideration, especially in older adults with other cardiovascular risk factors or complications already [9, 17].

In the case of our patient, it is postulated that his dysphagia was likely multifactorial in nature, given there was some degree of aspiration noted in the oropharyngeal phase by the SLP evaluator and on the video swallow fluoroscopy, which appeared to be independent of the globus sensation he experienced with food catching in his chest. Fortunately, our patient did demonstrate some improvement with modifications to diet and coaching on swallowing techniques, which led to a reduction in the observed aspiration in follow-up SLP evaluations. Consequently, we felt cardiothoracic surgery’s decision to forego a potentially very invasive surgery in a patient with advanced age and comorbidities in favor of conservative management was appropriate.

## Conclusions

Dysphagia is a common condition with increasing frequency in the older adult population, with significant associated morbidity apart from the impact on patients’ quality of life and nutritional status. Given the prevalence and an ever-aging population, it is important for screening and recognition in the outpatient primary care setting in order for earlier intervention. Furthermore, an awareness of rare causes of dysphagia, such as dysphagia lusoria, is imperative to ensure that timely and appropriate diagnostic testing is completed in order to allow appropriate multidisciplinary management.
